# Meteoroid Fragmentation in the Martian Atmosphere and the Formation of Crater Clusters

**DOI:** 10.1029/2021JE007149

**Published:** 2022-07-20

**Authors:** G. S. Collins, E. L. Newland, D. Schwarz, M. Coleman, S. McMullan, I. J. Daubar, Katarina Miljković, Tanja Neidhart, Eleanor Sansom

**Affiliations:** ^1^ Department of Earth Science and Engineering Imperial College London UK; ^2^ Earth, Environmental, and Planetary Sciences Brown University Providence RI USA; ^3^ Curtin University Bentley WA Australia

## Abstract

The current rate of small impacts on Mars is informed by more than one thousand impact sites formed in the last 20 years, detected in images of the martian surface. More than half of these impacts produced a cluster of small craters formed by fragmentation of the meteoroid in the martian atmosphere. The spatial distributions, number and sizes of craters in these clusters provide valuable constraints on the properties of the impacting meteoroid population as well as the meteoroid fragmentation process. In this paper, we use a recently compiled database of crater cluster observations to calibrate a model of meteoroid fragmentation in Mars' atmosphere and constrain key model parameters, including the lift coefficient and fragment separation velocity, as well as meteoroid property distributions. The model distribution of dynamic meteoroid strength that produces the best match to observations has a minimum strength of 10–90 kPa, a maximum strength of 3–6 MPa and a median strength of 0.2–0.5 MPa. An important feature of the model is that individual fragmentation events are able to produce fragments with a wide range of dynamic strengths as much as 10 times stronger or weaker than the parent fragment. The calibrated model suggests that the rate of small impacts on Mars is 1.5–4 times higher than recent observation‐based estimates. It also shows how impactor properties relevant to seismic wave generation, such as the total impact momentum, can be inferred from cluster characteristics.

## Key Points

- Crater cluster observations used to calibrate model of meteoroid fragmentation in Mars' atmosphere
- Fragments exhibit a wide range of dynamic strengths both weaker and stronger than parent
- The rate of small impacts on Mars is 1.5–4 times higher than recent observation‐based estimates

## 1 Introduction

Repeated imaging of the surface of Mars by orbiting spacecraft over the last two decades has revealed more than one thousand impact sites formed in this time period (Daubar et al., 2013, 2019, 2022). These observations provide important constraints on the current rate of small impacts on Mars, which are valuable for calibrating crater production models (Daubar et al., 2013; Williams et al., 2014), assessing the impact hazard to spacecraft on Mars, and determining the ratio of primary to secondary crater production rates (Hartmann et al., 2018).

Among the known recent impact sites, fewer than half are single craters; the majority are fields of craters, known as crater clusters (Daubar et al., 2013, 2019, 2022; Neidhart et al., 2021). The size and separation of individual craters within these clusters suggest that they are formed due to atmospheric break up of the primary meteoroid into a collection of fragments that separate and strike the ground almost simultaneously (Artemieva & Shuvalov, 2001; Popova et al., 2003, 2007). The diversity of crater clusters, in terms of the spatial distributions and size‐frequency distributions of their craters, provide a unique opportunity to interrogate the processes of atmospheric entry and fragmentation, and potentially constrain properties of the impactor population (Daubar et al., 2019; Hartmann et al., 2018; Neidhart et al., 2021). Differences in small crater and crater cluster populations between surfaces of different ages may also help constrain historical variations in atmospheric density and pressure (Chappelow & Sharpton, 2005; Kite et al., 2014; Popova et al., 2003).

A database of 634 recently formed crater clusters was mapped and characterized in detail by Neidhart et al. (2021). This represents about 90% of the clusters in the updated catalog of new impact sites on Mars (Daubar et al., 2022); clusters not included were added to the catalog after we had completed portions of our analysis. Here, we augment this data set of clusters with a random sample of 90% (456) of new single craters from the same catalog. The resultant data set includes crater size and position information for all individual craters larger than one m in all detected clusters and single craters. From the cluster data, a number of cluster properties were derived that characterize the spatial distribution and size‐frequency distribution of craters within the cluster (Daubar et al., 2019; Neidhart et al., 2021).

On the basis that the present catalog of small impacts, while incomplete, is representative of recent crater and cluster production on Mars, here we use these quantitative cluster characteristics to calibrate a model of meteoroid fragmentation in Mars' atmosphere (Artemieva & Shuvalov, 2001). This allows us to constrain key model parameters and the distribution of meteoroid properties such as dynamic strength. The calibrated model provides insight into the small impactor flux at Mars and how this compares to the observed flux at Earth (Bland & Artemieva, 2006). It also shows how impactor properties such as the total impact momentum can be inferred from cluster characteristics.

## 2 Modeling Meteoroid Fragmentation in the Martian Atmosphere

Several semi‐analytical atmospheric disruption models have been developed to simulate the passage and fragmentation of meteoroids through a planetary atmosphere (e.g., Artemieva & Shuvalov, 2016; Chyba et al., 1993; Herrick & Phillips, 1994; Hills & Goda, 1993; Korycansky & Zahnle, 2005; Passey & Melosh, 1980; Register et al., 2017). These can be broadly categorized based on their treatment of fragmentation. Continuous fragmentation models treat the fragmented meteoroid as a single, continuously deforming structure (e.g., Chyba et al., 1993; Hills & Goda, 1993). They are well suited to describing airbursts where the meteoroid experiences catastrophic break‐up into many small fragments, leading to a dramatic increase in drag and deposition of energy in the atmosphere (McMullan & Collins, 2019). Discrete fragmentation models, on the other hand, approximate fragmentation as a successive division of the meteoroid into individual masses. Such models can track the resulting fragments until complete ablation or impact (e.g., Artemieva & Shuvalov, 2001; Bland & Artemieva, 2006; Passey & Melosh, 1980; Popova et al., 2003). They are, therefore, ideally suited to modeling the formation of crater clusters or strewn fields formed by near simultaneous impact of a population of meteoroid fragments (Bland & Artemieva, 2006; Passey & Melosh, 1980; Popova et al., 2003). A third, hybrid approach that combines elements of both models has recently proved successful at replicating the energy deposition of a number of small terrestrial bolides (Register et al., 2017; Wheeler et al., 2017, 2018).

Here we apply a version of the discrete fragmentation model often referred to as the Separate Fragments Model (SFM) of atmospheric break‐up (Artemieva & Shuvalov, 2001; Bland & Artemieva, 2006; Passey & Melosh, 1980) to the formation of small crater clusters on Mars. This model was previously applied to the formation of crater strewn fields (clusters) on Earth by the break‐up of strong iron meteorites (Artemieva & Shuvalov, 2001; Bland & Artemieva, 2006; Passey & Melosh, 1980) and used to show that small crater clusters on Mars are consistent with the break‐up of much weaker stony meteoroids in Mars' more tenuous atmosphere (Artemieva & Shuvalov, 2001; Ivanov et al., 2009; Popova et al., 2003, 2007). Variants of this model were also used to simulate the formation of crater fields (clusters) on Venus and Titan (Herrick & Phillips, 1994; Korycansky & Zahnle, 2005). Similar models have also been used to understand the influence of atmospheric variations on the production of small craters on Mars (Chappelow & Sharpton, 2005; Williams et al., 2014). The availability of new observational data on the frequency and characteristics of small clusters and single craters provides the opportunity for a rigorous calibration of these models from which new insight into the rate and nature of small impacts on Mars can be derived.

### 2.1 Flight Integration

The basis for the SFM used here (Schwarz et al., 2022) is the coupled set of ordinary differential equations of standard meteor physics (e.g., Baldwin & Sheaffer, 1971; Passey & Melosh, 1980), which describe the temporal evolution of the meteoroid speed *v*, mass *m*, trajectory angle to the horizontal *θ* and position in space *x*, *y*, *z*:

(1) $$\frac{dv}{dt}=-\frac{C_{D}\rho_{a}v^{2}\pi r^{2}}{2m}+g\sin\left(\theta \right),$$

(2) $$\frac{dm}{dt}=-\frac{\sigma \rho_{a}v^{3}\pi r^{2}}{2},$$

(3) $$\frac{d\theta }{dt}=\frac{g\cos\left(\theta \right)}{v}-\frac{C_{L}\rho_{a}\pi r^{2}v}{2m}-\frac{v\cos\left(\theta \right)}{R_{p}+z},$$

(4) $$\frac{dz}{dt}=-v\sin\left(\theta \right),$$

(5) $$\frac{dx}{dt}=v\cos\left(\theta \right)\cos\left(\phi \right)\frac{R_{p}}{R_{p}+z},$$

(6) $$\frac{dy}{dt}=v\cos\left(\theta \right)\sin\left(\phi \right)\frac{R_{p}}{R_{p}+z},$$

(7) $$\frac{d\phi }{dt}=0.$$

In these equations, *C*_*D*_ and *C*_*L*_ are dimensionless coefficients of drag and lift, respectively, *σ* is an ablation parameter, *r* is the meteoroid radius (assumed to have a circular cross‐section orthogonal to the trajectory), *ρ*_*a*_ is the air density, *R*_*p*_ is the planetary radius, and *g* is the gravitational acceleration, which is a function of altitude *z*. The introduction of the azimuth *ϕ* and two coordinates for downrange distance *x* and cross‐range distance *y* allows the calculation of impact locations on a two‐dimensional planetary surface.

These equations are integrated numerically from a specified initial state with respect to time for the initial meteoroid and any fragment subsequently produced until the meteoroid or fragment (a) strikes the ground; (b) ablates to a size sufficiently small to be neglected; or (c) deflects off the atmosphere back into space. The form of the equations adopted here account for the effects of drag, lift, ablation, planetary curvature and the decrease of gravity with altitude. An atmospheric density‐altitude table extracted from the Mars Climate Database http://www-mars.lmd.jussieu.fr (Forget et al., 1999; Millour et al., 2018) at a point on the equator was used to provide a reference atmospheric density profile. The database provides meteorological fields, including density, compiled from General Circulation Models of the martian atmosphere, calibrated with available observational data. The specific table used is included in Collins et al. (2022). Altitude is defined relative to the MOLA zero altitude. As the density profile is approximately exponential and the density table is relatively coarse, intermediate density points were calculated using exponential interpolation.

### 2.2 Fragmentation and Separation

During integration of the meteor equations each meteoroid or fragment is assigned a dynamic strength *Y*. A widely used fragmentation criteria is then applied that the fragment breaks when the ram pressure (the product of the local air density and the fragment velocity squared) exerted on the meteoroid exceeds the dynamic strength *Y*:

(8) $$\rho v^{2}>Y.$$

In the version of the Separate Fragments Model used here, the parent meteoroid is always broken into two child fragments with a mass ratio chosen at random between specified limits. The two fragments are each assigned a strength, based partly on mass, and a relative velocity that acts to separate the two fragments in opposing directions, perpendicular to the direction of flight of the parent and at a random azimuth *ϕ* to the parent trajectory. The magnitude of the lateral separation velocity *v*_*S*_ is defined by (Passey & Melosh, 1980):

(9) $$v_{S}=v\sqrt{C_{S}\frac{3}{2}\frac{r_{1}}{r_{2}}\frac{\rho_{a}}{\rho_{m}}}$$

where v is the along trajectory velocity, *C*_*S*_ is a dimensionless separation velocity coefficient, *ρ*_*a*_/*ρ*_*m*_ is the ratio of the atmospheric to fragment density and *r*_1_/*r*_2_ is the ratio of the larger to the smaller fragment radii (where spherical geometry is assumed). The lateral velocity imparted to each fragment is determined so as to satisfy two constraints: linear momentum conservation and a net separation velocity of $v_{s}$.

The masses of the two child fragments are assigned randomly at each fragmentation event as a fraction of the parent fragment mass *m*_*p*_. The first fragment is assigned a mass equal to:

(10) $$m_{1}=m_{p}\left(1-x\right)f_{m},\;x∼U\left[0,f_{r}\right],$$

where *f*_*m*_ represents the nominal mass fraction of the first fragment and *f*_*r*_ is a randomization factor (where *f*_*r*_ = 0 implies *m*_1_/*m*_*p*_ is always *f*_*m*_ and *f*_*r*_ = 0.9 implies that the first fragment can take a mass fraction between 0.1*f*_*m*_ and *f*_*m*_). The second fragment is assigned the remaining mass *m*_2_ = *m*_*p*_ − *m*_1_.

The strength of each child fragment is determined based on two principles from previous implementations of the SFM (Artemieva & Shuvalov, 2001). First, we assume that in general the child fragment acquires an increased strength relative to the parent fragment owing to the removal of larger structural weaknesses and faults within the parent fragment. A second important principle is that the actual strength of the fragment is allowed to vary about this nominal value so that child fragments can, occasionally, be weaker than their parents. The strength *Y*_*f*_ of the fragment is therefore prescribed according to:

(11) $$Y_{f}=Y_{p}{\left(\frac{m_{p}}{m_{f}}\right)}^{\alpha }10^{x},\;x∼N\left(0,\delta \right).$$

The first term on the right‐hand side defines the nominal strength of the fragment based on Weibull statistics (Weibull, 1951), where *m*_*p*_ and *m*_*f*_ is the mass of the parent and child fragment, respectively, *Y*_*p*_ is the strength of the parent and *α* is the strength scaling exponent (Popova et al., 2011). A value of *α* close to zero implies little increase in strength as mass decreases. The second term on the right‐hand side is a random strength scaling factor drawn from a log‐normal distribution; that is, where the exponent *x* is drawn from a normal distribution with a mean of zero a standard deviation of *δ*.

A consequence of this random strength scaling factor, which turns out to be important for reproducing the characteristics of crater clusters, is that the child fragment can become either much stronger than its parent, potentially precluding any further fragmentation, or weaker than the parent, implying that the child fragment immediately undergoes at least one further fragmentation. This latter scenario effectively allows for more catastrophic fragmentation events, where the parent breaks into more than two fragments. This feature of the model, therefore, may be replicated by a model that splits the parent into a random number of fragments rather than always a pair. The resulting fragments are traced until either their mass is too small to produce a crater or an impact occurs.

### 2.3 Crater Formation and Cluster Characterization

Once fragments of the meteoroid strike the ground, we use a well‐established scaling relationship to determine the crater diameters *D* (Holsapple, 1993):

(12) $$\begin{array}{ccc} D & = & 2f_{\text{rim}}K_{r}V^{1/3},\\ \frac{\rho_{t}V}{m} & = & K_{1}{\left[\left(\frac{g_{0}r}{v_{z}^{2}}\right){\left(\frac{\rho_{t}}{\rho_{m}}\right)}^{\frac{6\nu -2-\mu }{3\mu }}+K_{2}{\left(\frac{Y_{c}}{\rho_{t}v_{z}^{2}}{\left(\frac{\rho_{t}}{\rho_{m}}\right)}^{\frac{6\nu -2}{3\mu }}\right)}^{\frac{2+\mu }{2}}\right]}^{-\frac{3\mu }{2+\mu }}. \end{array}$$

This relationship allows for a large range of potential surface properties and impact scenarios. *V* is the crater volume below the preimpact surface, *g*_0_ is the gravitational acceleration at the impact location, *r*, *v*_*z*_, *m* and *ρ*_*m*_ are the radius, vertical impact velocity component, mass and density of the impacting fragment, respectively. The factor *f*_rim_ = 1.3, converts the crater diameter at pre‐impact surface level to the value at the rim level to be consistent with observational measurements (Holsapple, 1993).

Based on the sizes of craters formed by mass‐balance devices released during the descent of Perseverance (Fernando et al., 2021), we modeled the martian surface with properties appropriate for a weak granular soil: density *ρ*_*t*_ = 1500 kgm^−3^, cohesive strength *Y*_*c*_ = 50 kPa, and with scaling coefficients *K*_1_ = 0.133, *K*_2_ = 1, *K*_*r*_ = 1.25, *μ* = 0.41, *ν* = 0.4 (Holsapple, 1993).

Due to the current resolution of satellite imagery, craters with a diameter smaller than 1 m cannot be resolved, therefore no craters below this limit are included in the observational data. To take this into account in the model applied here, small fragments that will not produce a crater larger than 1 m after accounting for overlap are removed from the model and not included in the results.

In cases where craters formed by two or more fragments overlap, they are combined into a single crater with a volume equal to the sum of the two crater volumes (justified by the approximately linear scaling between impactor mass and crater volume, Equation 12). Based on the proximity of craters in observed clusters (Daubar et al., 2019), the overlap criterion used here is that the separation distance of crater centers,

(13) $$d_{\text{sep}}<0.75\;max\left(R_{1},R_{2}\right)+0.25\left(R_{1}+R_{2}\right),$$

where *R*_1_ and *R*_2_ are the rim radii of the two craters. This criterion is a weighted combination of two thresholds: *d*_sep_ < max(*R*_1_, *R*_2_) implies that two craters are only merged if one of their centers lies within the other crater; *d*_sep_ < (*R*_1_ + *R*_2_) implies that two craters are merged when they touch.

For each cluster, a number of characteristics were calculated based on the sizes and spatial distribution of the craters (Neidhart et al., 2021). These characteristics include the number of craters *N*_*c*_ and the effective diameter,

(14) $$D_{\text{eff}}=\sqrt[{3}]{\sum_{i}D_{i}^{3}}.$$

Effective diameter is proportional to the cube root of the total volume of all craters in the cluster and, hence, is a measure of the equivalent crater size if fragmentation did not occur (Daubar et al., 2013; Malin et al., 2006).

To quantify the spatial distribution of a cluster, we define the dispersion of the cluster as the median separation of all crater pairs *d*_med_ (Neidhart et al., 2021). For clusters with more than five craters, the aspect ratio of the cluster *e* is defined as the ratio of the semi‐minor to semi‐major axis of the ellipse of minimum area that encompasses the location of the majority of craters in the cluster. The algorithm for determining the best‐fit ellipse is described in detail by Daubar et al. (2019). Our implementation of the algorithm is included in Collins et al. (2022). For a given cluster, the influence of outliers on the ellipse of best fit is minimized by performing many independent fits and taking the mean aspect ratio of all ellipses. Each fit uses a sample of craters (of the same number as in the observed cluster) drawn with equal probability and with replication from the observed distribution. Finally, to characterize the size‐frequency distribution of craters within the cluster, we calculate several metrics: the maximum, median and minimum crater diameter (*D*_max_, *D*_med_, *D*_min_); the number and fraction of craters in the cluster with diameter larger than half the maximum diameter (*N*(>*D*_max_/2), *f*_*L*_ = *N*(>*D*_max_/2)/*N*_*c*_); and the exponent of the power‐law that best fits the cumulative size‐frequency distribution of craters in the cluster (i.e., *γ*, in *N*(>*D*) ∝ *D* ^−*γ*^). This exponent *γ* was determined by performing a linear regression between log  *N*(>*D*) and log  *D* for clusters with more than five craters.

**Figure 1:**
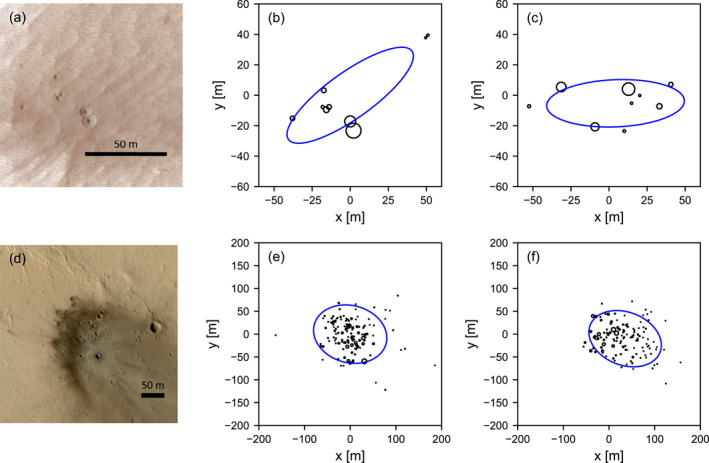
(a) HiRISE image of a crater cluster on Mars (ESP_037706_1765; NASA/JPL/University of Arizona) with number of craters *N*_*c*_ = 9, effective diameter *D*_eff_ = 11.4 m, aspect ratio *e* = 0.3, dispersion *d*_med_ = 27.2 m (b) Digitized version of the same crater cluster where each circle represents a single crater to scale. (c) An example of a comparable model crater cluster produced by the Separate Fragments Model (SFM) with *N*_*c*_ = 9, *D*_eff_ = 10.1 m, *e* = 0.34, *d*_med_ = 33.7 m (d) HiRISE image of a crater cluster on Mars (ESP_038458_2030; NASA/JPL/University of Arizona) with *N*_*c*_ = 133, *D*_eff_ = 16.1 m, *e* = 0.77, *d*_med_ = 55.8 m. (e) Digitized version of the same crater cluster where each circle represents a single crater to scale. (f) An example of a comparable model crater cluster produced by the SFM with *N*_*c*_ = 120, *D*_eff_ = 15.7 m, *e* = 0.71, *d*_med_ = 65 m. In the simulated crater clusters the impact trajectory direction is from left to right. Note that the ellipses (blue) are fit to the data using the method described in (Daubar et al., 2019). Note also the occurrence of some pre‐existing craters in (d) that are not part of the crater cluster.

Figure 1 shows two examples of observed crater clusters on Mars, together with a digitized representation of the same clusters based on mapping of Daubar et al. (2019). It also displays examples of clusters produced by the SFM used in this work that are comparable in terms of all characteristics, including the number of craters *N*_*c*_, effective diameter *D*_eff_, dispersion *d*_med_ and aspect ratio *e*.

### 2.4 Monte Carlo Modeling

While the ability of the SFM to reproduce individual clusters is reassuring, a rigorous test of model performance requires that that model can replicate the statistical distribution of observed crater clusters as well as the proportion of events that result in formation of a cluster rather than a single crater.

To produce a set of synthetic crater clusters for comparison with those observed on the surface of Mars, a number of Monte Carlo simulations were carried out. While some observed cluster characteristics, such as effective diameter and number of craters, show a dependency on surface elevation (Neidhart et al., 2021), the ratio of cluster‐forming to single‐crater impacts is independent of surface elevation (Daubar et al., 2022) and about 50% of impact sites have surface elevations within 2 km of the MOLA 0 km reference. For this study, therefore, all simulations used the same atmospheric density profile, with a surface air density of 0.0157 kg m^−3^, a surface elevation of 0 km and began with the meteoroid at an altitude of 100 km. Future work will investigate the sensitivity of cluster characteristics to variation in ground elevation and surface air density, which varies by a factor of ∼2 across Mars and by more than an order of magnitude during Mars' history (Chappelow & Sharpton, 2005). We also adopted the same drag coefficient *C*_*D*_ = 1 and nominal fragment mass fraction *f*_*m*_ = 0.5, throughout.

All other initial properties of the meteoroid were selected at random or according to parameter probability distributions for Mars. The probability distribution for pre‐entry velocity was derived from Le Feuvre and Wieczorek (2011) and we used the canonical probability distribution for impact angle *P*(>*θ*) = sin^2^ *θ* (Shoemaker, 1961). Following Williams et al. (2014), meteoroid mass was drawn from a Pareto distribution based on observations of terrestrial fireballs for meteoroids >3 kg (Bland & Artemieva, 2006; Halliday et al., 1996); $P\left(>m\right)={\left(m/m_{\text{min}}\right)}^{-0.926}$, where *m*_min_ is the minimum mass meteoroid mass in the simulation. For most simulations presented here a minimum mass of 15 kg was used as a compromise between computational expedience and generating a statistically complete synthetic data set for craters or clusters with an effective diameter greater than 10 m.

To select meteoroid density, we used a uniform probability distribution between lower and upper bounds of 1400 and 4000 kg m^−3^, respectively. We adopted this simple approach because the real distribution of meteoroid bulk densities is not well known. The density distribution of common stony meteorites is well established (Britt & Consolmagno, 2003), and while bulk meteoroid densities will be lower because of macroporosity, how the density of the surviving fragment maps to pre‐entry bulk meteoroid density is not well constrained, particularly for underrepresented meteorite types. Although they may represent a substantial proportion of all meteoroids (Ceplecha et al., 1998; Halliday et al., 1996), we do not consider meteoroids with density less than 1400 kg m^−3^, including cometary meteoroids, because in our size range of interest these objects rarely survive passage through the atmosphere to form craters on the ground larger than 1 m (Williams et al., 2014). We also omit the small population (≤5% by mass Bland & Artemieva, 2006; Ceplecha et al., 1998) of iron meteoroids, which are strong enough that their fate is either to ablate entirely or impact the ground as a single fragment and form a single crater (Popova et al., 2003). Trial simulations using a density, ablation parameter and strength range appropriate for iron meteoroids confirmed that 12% of scenarios (with mass >15 kg) resulted in airburst or no crater larger than 1 m (the same as for stony meteoroids) and ≤1% resulted in a crater cluster for a nominal minimum iron meteoroid strength of 3 MPa. Iron meteoroids are therefore not expected to make an important contribution to the population of crater clusters on Mars. Moreover, neglecting iron meteorite scenarios will therefore only overestimate the rate of cluster formation by a small factor.

The probability distribution of the dynamic strength of meteoroids appropriate for our fragmentation criterion (Equation 8) is also not well known. As this parameter is of critical importance for the frequency of cluster formation and the characteristics of clusters, we treat this distribution as one of the principal unknowns in our analysis. For simplicity, we adopt a log‐uniform probability distribution, defined by a median dynamic strength *Y*_med_ and a width (in log_10_ *Y*‐space) of *w*. In other words, the log‐uniform distribution has a minimum dynamic strength of *Y*_min_ = *Y*_med_10^−*w*^ and a maximum dynamic strength *Y*_max_ = *Y*_med_10^*w*^.

The ablation parameter *σ* is another meteoroid property that is not well constrained. Here we select this parameter from a uniform probability distribution between the lower and upper bounds for stony meteorites (1 × 10^−8^–4.2 × 10^−8^ s^2^ m^−2^, respectively; Ceplecha et al., 1998) and neglect any correlation with density, size or velocity (Baldwin & Sheaffer, 1971; Brykina & Bragin, 2020). Model parameters that we vary to determine the most appropriate for replicating Mars crater clusters are the strength‐mass scaling exponent *α* and randomization factor *δ* (Equation 11), the lift coefficient *C*_*L*_ (Equation 3), the fragment mass fraction randomization factor *f*_*r*_ (Equation 10) and the fragment separation coefficient *C*_*S*_ (Equation 9). The ranges of these parameters explored in our simulations are shown in Table 1.

**Table 1:** Best‐Fit Values and Variation Range for Model Parameters

Parameter	Baseline value	Variation range
Initial density, *ρ*_*m*_	‐	1400–4000 kg m^−3^
Ablation parameter, *σ*	‐	1–4.2 × 10^−8^ s^2^ m^−2^
Median initial strength, *Y*_med_	330 kPa	100–1000 kPa
Width of strength distribution, *w*	1.25	0.5–1.5
Lift coef., *C*_*L*_	0.02	0.005–0.05
Separation velocity coef., *C*_*S*_	0.6–2.2	0.1–2.2
Strength‐mass scaling exponent, *α*	0.25	0.01–0.8
Strength‐mass scaling variance, *δ*	0.5	0–1
Fragment split coef., *f*_*r*_	0.9	0–0.99

To determine the optimum meteoroid and model parameters or parameter distributions that produce a synthetic set of crater clusters (and single craters) most consistent with observations (Daubar et al., 2022; Neidhart et al., 2021), we used a three‐phase approach. In a first exploratory phase, we performed a number of experiments with different parameter/parameter distribution combinations to determine good parameter combinations for more detailed study. In this phase, each Monte Carlo simulation was run until the synthetic crater population included 200 singular craters or crater clusters with an effective diameter greater than 10 m. This target was adopted as it is the approximate number of such craters in the observational data set and is less sensitive to biases in the observational data (Daubar et al., 2022; Neidhart et al., 2021).

Agreement between the observed distribution of single craters and crater clusters and the simulation results was assessed based on both a quantitative comparison of the frequency distributions of individual cluster characteristics (e.g., *D*_eff_, *N*_*c*_) and a qualitative comparison of two‐dimensional frequency distributions in various characteristic‐spaces (e.g., *D*_eff_‐*N*_*c*_).

A quantitative comparison of the model frequency distribution of a given cluster characteristic with the observed distribution was made by binning the distribution and calculating the chi‐square statistic of the binned distributions, which quantifies the mismatch between the two binned distributions. For example, for the number of craters characteristic, *N*_*c*_, we first bin the observed (normalized) frequency distribution of this characteristic to define $F_{i}^{o}\left(N_{c}\right)$ using 10–15 bins, depending on the characteristic. We then bin the model frequency distribution using the same bins to define $F_{i}^{m}\left(N_{c}\right)$. The chi‐square statistic is then computed for this characteristic as:

(15) $$\chi^{2}\left(N_{c}\right)=\sum_{i}^{n}\frac{{\left[F_{i}^{m}\left(N_{c}\right)-F_{i}^{o}\left(N_{c}\right)\right]}^{2}}{F_{i}^{o}\left(N_{c}\right)}.$$

A more qualitative comparison between the observed distribution of cluster characteristics (Neidhart et al., 2021) and those produced by the model was made by visual inspection of kernel‐density plots of the distributions in various characteristic‐spaces (e.g., *D*_eff_‐*N*_*c*_, *e*‐*d*_med_). This provided confirmation that important correlations between cluster characteristics present in the observational data are replicated by the model.

As certain crater cluster characteristics require more than five craters for meaningful determination, we restricted our comparison to the 84% of observed clusters with *N*_*c*_ > 5. We also omit all clusters and craters with *D*_eff_ < 5.65 m ($4\sqrt{2}$ m) from our comparison to compromise between statistical power and likely completeness of the observational data set. An additional important statistic to determine model performance was the ratio of the total number of crater clusters to singular craters (C‐S ratio) with effective diameters >10 m produced by the model. The observed C‐S ratio is 1.68 for all clusters with *D*_eff_ > 10 m, 1.46 for clusters with *N*_*c*_ > 5 and *D*_eff_ > 10 m and 1.39 for the entire catalog (Daubar et al., 2022).

In the second phase of our approach, simulations using the most promising parameter combinations were performed with five times more samples to refine the best‐fit model parameters and quantify the sensitivity of model performance to the number of samples. Model performance was measured in the same quantitative and qualitative way. The best‐fit Monte Carlo model parameters from this phase are given in Table 1. In the final phase, several suites of simulations were performed whilst systematically varying key model parameters using orthogonal sampling. The purpose of this final phase was to confirm that the most‐promising model was a local optimum and to quantify the sensitivity of the model performance to variation of key meteoroid properties and model parameters.

For brevity and clarity of presentation, here we describe the results of the latter two of these phases: we first present the results of our best‐fit model; we then consider the sensitivity of model performance to the variation of influential individual model parameters.

## 3 Results

### 3.1 Best‐Fit Model

**Figure 2:**
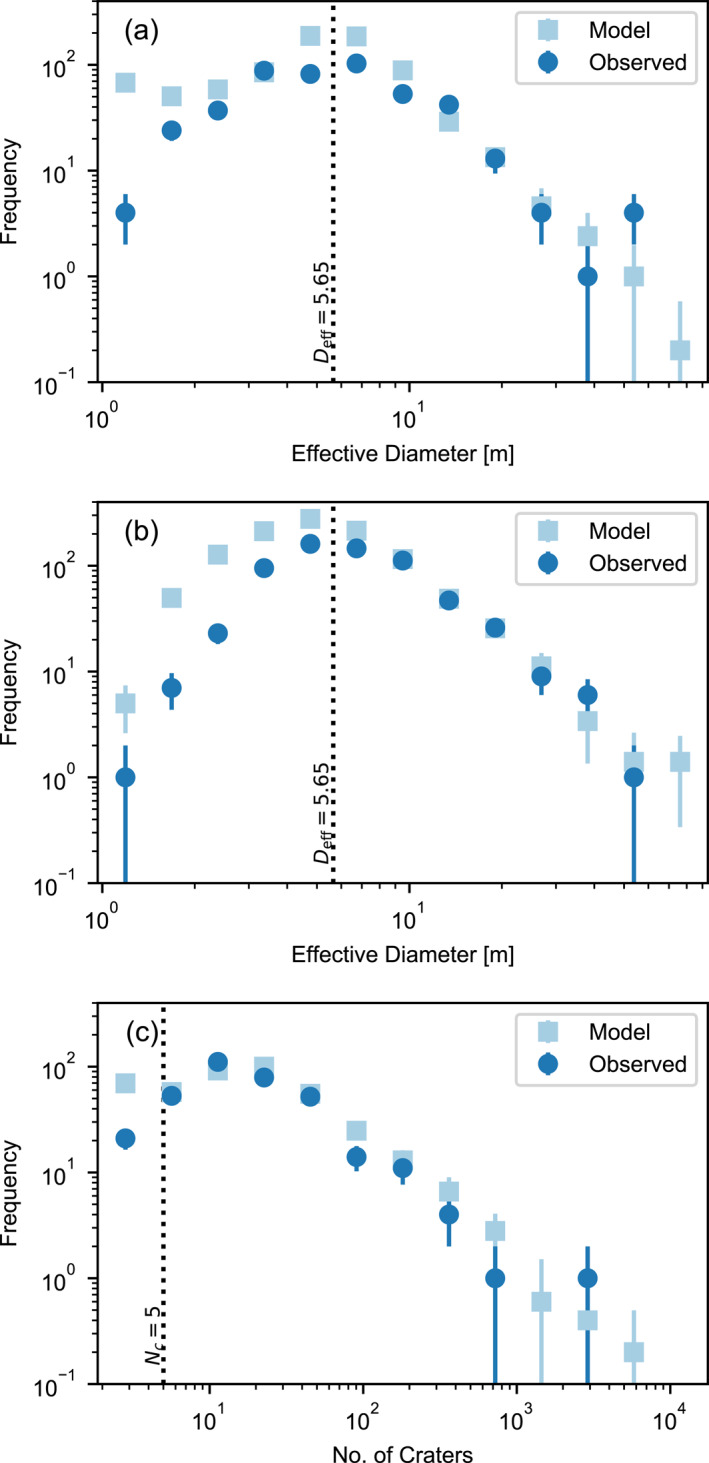
Incremental frequency distributions of (a) diameter of singular craters; (b) effective diameter of crater clusters (with *N*_*c*_ > 1); (c) number of craters in a cluster (with *D*_eff_ > 5.65) predicted by the best‐fit model compared with observations. Error bars for the models and observations are $\sqrt{N}$. The dotted lines indicate the threshold effective diameter (a and b) and number of craters in the cluster (c) above which the models and observations are quantitatively compared.

The best‐fit Monte Carlo simulation produced in this work used the best‐fit parameters listed in Table 1. The simulation produced a synthetic data set that includes 1000 craters/clusters with *D*_eff_ > 10 m. The incremental frequency distributions of (a) diameter of singular craters; (b) effective diameter of crater clusters (with *N*_*c*_ > 1); (c) number of craters in a cluster (with *D*_eff_ > 5.65) in this synthetic data set are compared with observations (Neidhart et al., 2021) in Figure 2 after scaling to the same population size (200 craters/clusters with *D*_eff_ > 10). The relative frequency of singular craters and clusters is very consistent with observations above 10 m in (effective) diameter, which is the threshold chosen as the most robust measure of the ratio of clusters to singular craters. The best‐fit model produces a cluster‐singular crater ratio of 1.62 for *D*_eff_ > 10 m and 1.35 for those clusters with *N*_*c*_ > 5 (*c*.*f*. observed values of 1.68 and 1.46, respectively).

Below an effective diameter of 5.65 m ($4\sqrt{2}$ m) there is a clear drop‐off in the observed number of craters and clusters, which is attributed to observational detection limitations (Daubar et al., 2022). The drop‐off in simulated crater/cluster numbers at approximately the same diameter is a consequence of the minimum meteoroid mass (15 kg) used in the models and not the effects of atmospheric filtering, which become most important for craters <1 m diameter (Williams et al., 2014).

Apart from a small excess of clusters with large numbers of craters, the simulation agrees well with the frequency distribution of *N*_*c*_ for *N*_*c*_ > 5. For *N*_*c*_ < 5 (and *N*_*c*_ = 2 specifically) the simulation predicts substantially more clusters than observed. This is likely a consequence of the frequency with which the SFM splits the meteoroid into only two fragments, with no further fragmentation events. The implications of this observation are discussed further in Section 4.1.

For these reasons, in the subsequent analysis we quantitatively compare the synthetic and observed distributions only for craters/clusters with an effective diameter larger than 5.65 m (with *D*_eff_ > 5.65) and for clusters with more than five craters (with *N*_*c*_ > 5).

**Figure 3:**
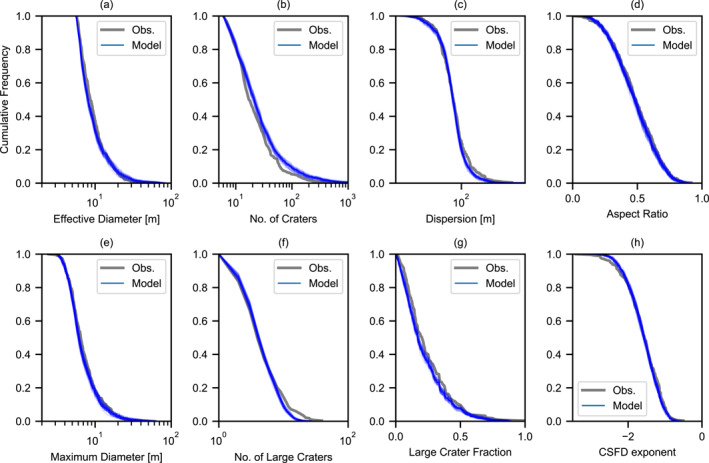
Normalized cumulative frequency distributions of various crater cluster characteristics for observed crater clusters on Mars (gray) and simulated crater clusters (blue). The CFDs are for all clusters with *N*_*c*_ > 5 and *D*_eff_ > 5.65 m. Dark blue curve represents the CFDs of all the clusters in the full synthetic data set, which comprises 1000 clusters/crater with *D*_eff_ > 10 m; light blue curves represent CFDs of 40 sub‐samples of the full data set with approximately the same number of *D*_eff_ > 10 m craters/clusters as observed (200).

The synthetic data set produced by the best‐fit Monte Carlo simulation has the lowest aggregated chi‐square statistic for all cluster characteristics in our investigation. The best‐fit model cumulative frequency distributions for eight cluster characteristics show an excellent agreement with the observed normalized frequency distributions (Figure 3). For almost all characteristics, small discrepancies are explainable by statistical variations. The thin blue curves in Figure 3 show 40 model frequency distributions obtained by sub‐sampling from the full synthetic data set to a sample size equivalent to the observational data set.

The model tends to produce clusters with a slightly larger number of craters than observed (Figures 2c and 3b) and with slightly fewer large craters than observed (Figure 3f). The latter discrepancy may suggest that a proportion of the meteoroid population produce stronger large fragments than tend to be produced by the SFM model used here. The former discrepancy, on the other hand, is likely to be at least in part because of uncertainty in crater scaling combined with the inability to resolve all the smallest craters in an observed cluster image (Daubar et al., 2019; Neidhart et al., 2021). A factor of two uncertainty in the cohesion of the martian surface translates to a ≈13% uncertainty in crater diameter (Equation 12). Increasing the minimum crater size threshold in the model by only 10%–20% (equivalent to one pixel on a HiRISE image) is sufficient to bring the model into agreement with the observations.

**Figure 4:**
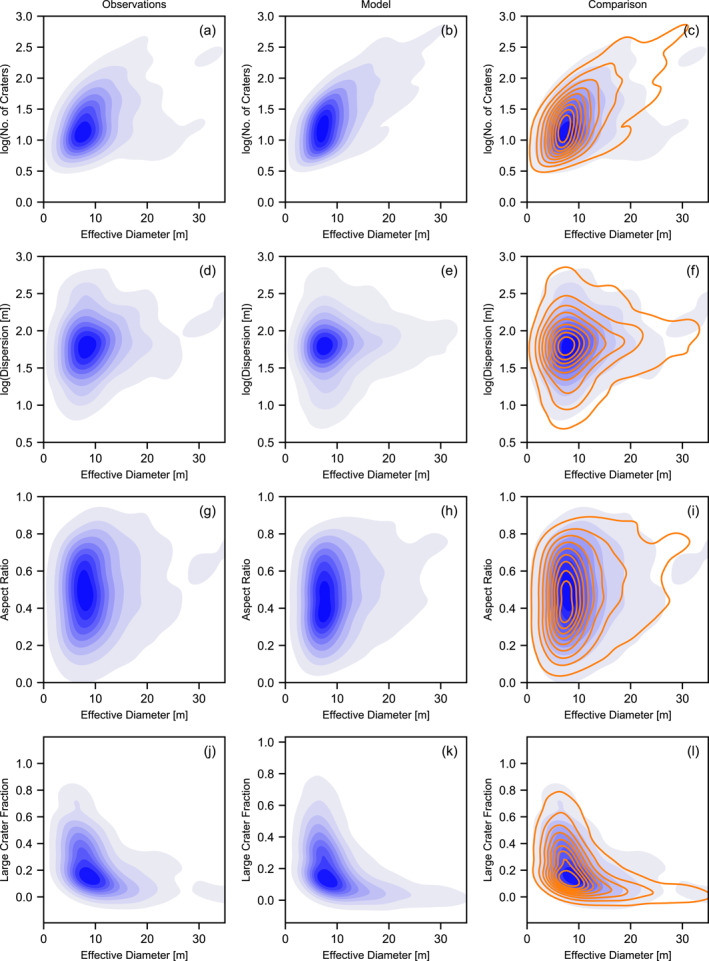
Kernel density comparison of various observed and simulated crater cluster characteristic distributions as a function of effective diameter *D*_eff_. In the third column, the observation distributions are shown in blue and the model distributions are shown in orange.

**Figure 5:**
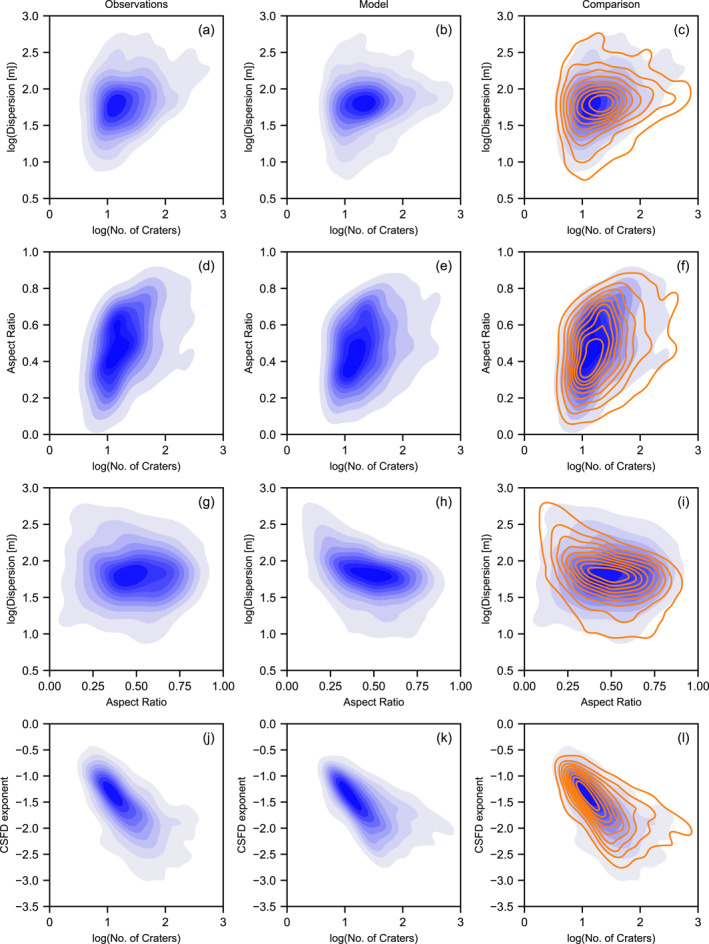
Kernel density comparison of various observed and simulated crater cluster characteristic distributions as a function of number of craters in the cluster *N*_*c*_ or aspect ratio. In the third column, the observation distributions are shown in blue and the model distributions are shown in orange.

As an additional test of the consistency between the simulation and observations, we compared two‐dimensional Kernel Density plots of the cluster characteristic distributions in various spaces (Figures 4 and 5). While this comparison does not provide a quantitative metric of mis‐fit, it visually confirms that correlations between observed characteristics are replicated by the Monte Carlo simulation and that the model does not produce clusters that are inconsistent with observations. In particular, the simulation replicates very well the variation in dispersion, number of craters, aspect ratio and large crater fraction among clusters with an effective diameter of 5–10 m, and how this variation changes as effective diameter increases. Also well replicated is the positive correlation between effective diameter and number of craters and the negative correlation between the large crater fraction *f*_*L*_ and effective diameter (Figure 4).

Similarly, the variation in dispersion, aspect ratio and the exponent of the cumulative size‐frequency distribution of craters in the cluster (*γ*) with increasing number of craters and the correlations between these characteristics are all very consistent between the simulated and observed clusters (Figure 5). One interesting trend that is present in the synthetic clusters, but not obviously apparent in the observational data is a negative correlation between dispersion and aspect ratio. In the synthetic data, clusters that are more circular tend to be slightly less dispersed than those clusters that are more elongated. This intuitive result occurs because shallower (and, hence, longer) trajectory impacts tend to produce more dispersed and elongated clusters. In the observed data, on the other hand, there is almost no correlation between dispersion and aspect ratio. This may suggest that other factors not taken into account in the model, such as differences in cluster elevation (atmospheric density), may exert a greater influence on dispersion than impact angle.

**Figure 6:**
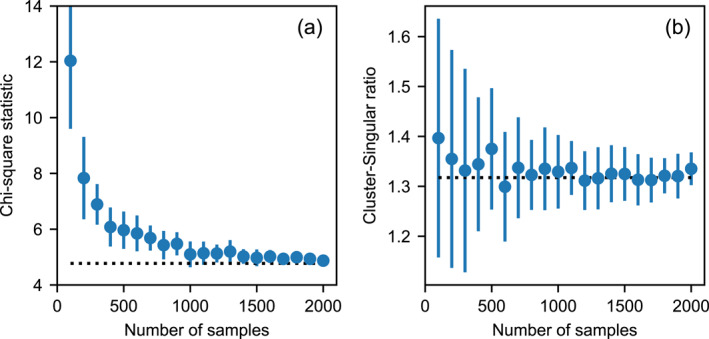
Convergence of the aggregate chi‐square statistic for all cluster characteristics (a) and cluster‐singular ratio (*N*_*c*_ > 5) (b) with increasing sample size. The number of samples on the *x*‐axis is the number of simulated impact events that produce a single crater or cluster with *D*_eff_ > 10 m (the equivalent sample size of observed clusters is approximately 200). The total number of impacts simulated is much larger.

The number of samples required for a robust assessment of the model was determined by aggregating the chi‐square statistic for the eight cluster characteristics shown in Figure 3 and analyzing the change in this quantity, as well as the C‐S ratio, with increasing sample size in the Monte Carlo simulation (Figure 6). For each sample size, multiple samples were drawn from the complete synthetic data set to quantify the variance in each statistic, shown as error bars in Figure 6. A synthetic sample size of 200 events with *D*_eff_ > 10 m, which is approximately equivalent to the observed sample size, is insufficient to give a robust comparison between the model and observations; however, a sample size of 1000 appears to be a good compromise between robustness and computational expedience. We note that as this sample size is greater than that of the observational data set, the 200‐sample variance of the C‐S ratio and chi‐square statistic are the most relevant measures of uncertainty between the simulation and observations for assessing model sensitivity.

### 3.2 The Influence of Meteoroid Properties and Model Parameters on Cluster Characteristics

Using the best‐fit Monte Carlo simulation as a baseline, we then explored the sensitivity of model performance to various influential meteoroid and SFM parameters using orthogonal sampling (i.e., running additional Monte Carlo simulations, varying one parameter while setting all other parameters as the baseline).

**Figure 7:**
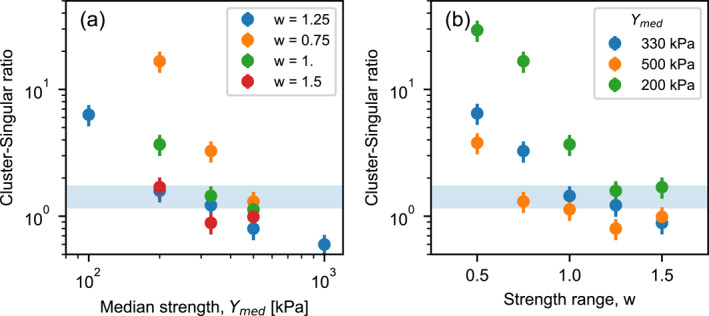
Cluster‐singular ratio (*N*_*c*_ > 5) as a function of (a) the median meteoroid strength *Y*_med_ (for a fixed width of the meteoroid strength distribution *w*); and (b) width of the meteoroid strength distribution *w* (for fixed median meteoroid strength *Y*_med_). Error bars represent the standard deviation in C‐S ratio from 40 200‐sample subsets of the best‐fit model (Figure 6). The light‐blue band indicates the observed cluster‐single ratio, including uncertainty.

The C‐S ratio is most sensitive to the initial dynamic strength of the meteoroid, which is governed in the SFM by the median strength *Y*_med_ and the strength range parameter *w* (Figure 7). Sensitivity analysis suggests that the optimum median strength is between 200 and 500 kPa and the width (in log‐space) of the log‐uniform distribution is between 0.75 and 1.5. This range of optimum meteoroid strength distribution parameters imply that in all scenarios consistent with observations the upper bound of the initial meteoroid strength distribution is 3–6 MPa, while the lower bound of the strength distribution ranges from 11 to 90 kPa.

**Figure 8:**
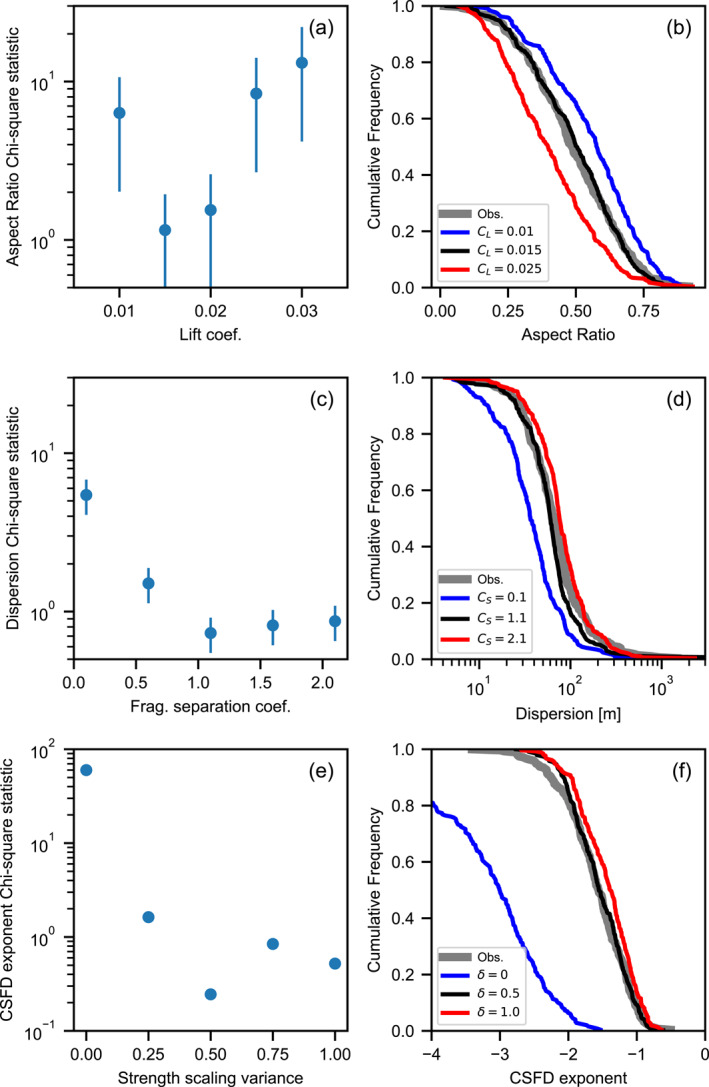
Chi‐square statistic (left) and cumulative frequency distributions (right) for three crater cluster characteristics as a function of the most influential fragmentation model parameter. Aspect ratio as a function of the lift coefficient *C*_*L*_ (a and b); dispersion as a function of fragmentation separation coefficient (c and d); and the exponent of the cumulative size‐frequency distribution of craters in the cluster (*γ*) as a function of the strength‐mass scaling parameter *δ* (e and f). Error bars in (a, c and e) represent the standard deviation in chi‐square statistic for the given cluster characteristic from 40 200‐sample subsets of the best‐fit model (Figure 6). Cumulative frequency distributions in (b, d and f) are for all clusters with *N*_*c*_ > 5 and *D*_eff_ > 5.65 m.

Optimization of the model also allows for the calibration of SFM parameters that are unknown or not well constrained. The fit to cluster aspect ratio *e* is sensitive to the lift coefficient *C*_*L*_ (Figures 8a and 8b). A lift coefficient that is too low leads to clusters that are too circular on average, while a lift coefficient that is too high leads to too many highly elongated clusters compared with observations. To minimize the chi‐square statistic for cluster aspect ratio requires a lift coefficient of 0.015–0.02.

The fit to dispersion (*d*_med_, the median separation between all crater pairs in a cluster) is sensitive to the fragment separation coefficient *C*_*S*_ (Figures 8c and 8d). A *C*_*S*_ coefficient that is too low results in crater clusters that are not as dispersed in space as observed and vice‐versa. To minimize the chi‐square statistic for dispersion appears to require a *C*_*S*_ > 1, although a range of values give equally good performance.

The exponent of the cumulative size‐frequency distribution of craters in the cluster (*γ*) is influenced most sensitively by the strength‐mass scaling parameter *δ*, which dictates the variation in child fragment strength relative to its parent (Figures 8e and 8f). In particular, the model performs very poorly for *δ* = 0, which implies that the child fragment strength is always stronger than the parent by a factor related to the child/parent mass ratio. In this case, the clusters tend to have crater size‐frequency distributions that are too steep on average, with a median *γ* ≈ 3. If *δ* is too large, on the other hand, the clusters tend to have crater size‐frequency distributions that are too shallow. To minimize the chi‐square statistic for *γ* we found *δ* ≈ 0.5, although a range of similar values give good performance. This value of *δ* implies that the random variation in child fragment strength should be such that there is a 95% chance the fragment strength is within a factor of 10 times lower than or higher than the nominal (mass‐dependent) value.

**Figure 9:**
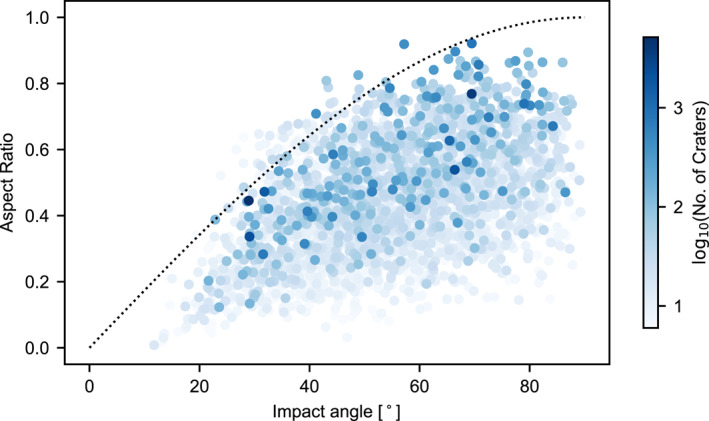
Aspect ratio *e* of the best‐fit ellipse that encompasses most craters in the cluster as a function of initial meteoroid entry impact angle to the horizontal *θ*. Results shown are for the best‐fit model described in the text and colored by log_10_ *N*_*c*_. The dotted line *e* = sin *θ* is a simple geometric model described in the text.

The aspect ratio of the best‐fit ellipse that encompasses most craters in the cluster has been proposed as a proxy for impact angle (Daubar et al., 2019). A simple geometric model that projects a cloud of meteoroid fragments that is axially symmetric about the main trajectory onto the surface to produce the cluster predicts a cluster aspect ratio *e* = sin *θ*. However, while our model results confirm that aspect ratio correlates with impact angle, particularly for clusters with many craters, impacts at a given angle can produce clusters with a wide range of aspect ratios (Figure 9). Our results suggest that the simple geometric projection model is not a good predictor of impact angle, but does provide a reasonable lower bound for the range of possible impact angles that could have formed the cluster. In other words, the observed cluster aspect ratio *e* can be used to preclude an impact angle less than sin^−1^ *e*.

While the impact trajectory direction is not known for the observed crater clusters, our simulated clusters show that in general the orientation of the semi‐major axis of the best‐fit ellipse is a good proxy for trajectory direction, especially for more elongated (low aspect ratio) clusters. The mean azimuth of the semi‐major axis of the best‐fit ellipse is within 2° of the trajectory line for the simulated clusters. The standard deviation of this azimuth, which is a measure of the uncertainty in relating the semi‐major axis of the best‐fit ellipse to impact direction, is 26° for all simulated clusters (with *N*_*c*_ > 5), 38° for clusters with *e* > 0.7 and 13° for clusters with *e* < 0.3. We also note that in both the observed and simulated clusters the largest crater in the cluster is most commonly located within the middle half of the ellipse and not near either end. This is contrary to observed crater fields on Venus where the largest crater is typically the furthest downrange (Herrick & Phillips, 1994).

### 3.3 Meteoroid Properties as a Function of Outcome

**Figure 10:**
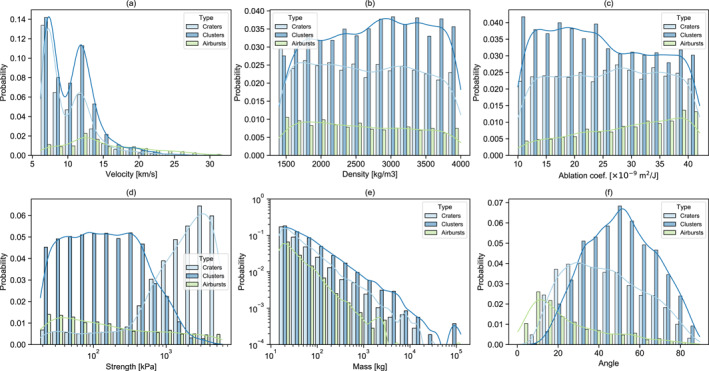
Probability distributions of meteoroid properties by outcome. Histograms of the relative frequency with which individual meteoroid properties result in a single crater, a crater cluster (meteoroid fragments in the atmosphere to form multiple craters) and an airburst (meteoroid is entirely ablated or forms no crater larger than 1‐m diameter on the ground).

The calibrated and optimized SFM provides insight into the probability distribution of initial meteoroid properties in the 15–10,000 kg mass range as a function of outcome (Figure 10). In the synthetic data set produced by the best‐fit model, crater cluster is the dominant outcome for meteoroid strengths less than 650 kPa, consistent with previous estimates (Williams et al., 2014), while single crater formation dominates for stronger meteoroids. However, it is evident that some weak meteoroids can still produce a single crater and meteoroids as strong as 3 MPa will occasionally fragment to produce a cluster.

Airbursts, which represent about 12% of the impacts simulated, are slightly more likely among low‐strength meteoroids. Among the other meteoroid properties, favorable parameters for airburst are high velocity, high ablation parameter, low mass and shallow angle impacts. Favorable scenarios for crater clusters are steeper angle impacts, with low ablation parameter and moderate‐to‐low entry velocity, while formation of a single crater is favored by impacts at intermediate angles and low entry speeds. In the meteoroid mass range considered in our model the relative proportion of clusters craters and airbursts is relatively independent of mass; however, extending the model to lower masses would result in a transition to a predominance of single craters and then a predominance of airbursts (Popova et al., 2003; Williams et al., 2014).

## 4 Discussion

### 4.1 Constraints on Meteoroid Properties and SFM Parameters

The dynamic meteoroid strength distribution used in our work that produces a synthetic cluster population that best matches the observed population is a log‐uniform distribution with a minimum strength of 10–90 kPa, a maximum strength of 3–6 MPa and a median strength of 0.2–0.5 MPa. This strength range is very consistent with the inferred dynamic strengths of stony meteoroids observed to disrupt in Earth's atmosphere (e.g., Borovička et al., 2019, 2020; Ceplecha et al., 1998; Popova et al., 2011; Wheeler et al., 2018). It is possible that other strength distributions may produce a cluster population that is a similar or even better fit to observations, but they would have to have the same (approximate) minimum, median and maximum values.

Investigating alternative strength distributions would be a fruitful avenue for further work. In particular, evidence from terrestrial meteoroid entry observations suggests that the strength distribution and fragmentation behavior of carbonaceous chondrites and ordinary chondrites may differ (Borovička et al., 2020). Carbonaceous chondrite meteoroids tend to exhibit a low initial dynamic strength of ∼50 kPa and then undergo a relatively continuous sequence of fragmentation events until deceleration is complete (Borovička et al., 2019). Ordinary chondrites, on the other hand, often exhibit a two‐stage fragmentation process, which may suggest that these meteoroids possess a bi‐modal internal strength (Borovička et al., 2020). While most such meteoroids experience an initial break‐up at a dynamic strength of ∼100 kPa, for a substantial proportion of events there is then a hiatus in fragmentation until the ram pressure exceeds ∼1 MPa. This has been interpreted as evidence that many ordinary chondrite meteoroids possess two strengths: a very low bulk strength that may be related to weak cementation of fragments and a stronger internal strength that is required to break the (partially fragmented) fragments themselves (Borovička et al., 2020).

It is possible that a bi‐modal meteoroid strength distribution may improve the agreement between model results and observations. Fewer meteoroids with an intermediate strength may reduce the number of meteoroids that fragment only once to produce two craters, which is a clear deficiency of the model (Figure 2). Similarly, this may also reduce the number of clusters with a large number of craters without at the same time reducing the proportion of meteoroids that produce clusters compared with singular craters, producing a better match to the observations. Modifying the SFM to account for a two‐phase fragmentation process may also improve the model in regards to the number of large craters within the cluster. This could be achieved, for example, by representing the meteoroid with two structural components (e.g., Borovička et al., 2020; Wheeler et al., 2018)—a weaker component that represents the fragile bonding of the bulk of the meteoroid and a stronger component that represents large fragments within the meteoroid that contain internal fractures but are more‐or‐less intact. Such a meteoroid would undergo one phase of fragmentation at high altitude to form a well‐separated cluster, and a second fragmentation phase much closer to the ground that would tend to produce larger craters.

Apart from the dynamic strength distribution of impacting meteoroids, the success of the model employed here in replicating cluster formation on Mars provides insight into several important aspects of the model and its parameters. Given the simplicity of the discrete fragmentation model with pair‐wise separation that we employ, the excellent agreement with observations suggests that more elaborate models may not be necessary to explain atmospheric break‐up of stony meteorites on Mars. On the other hand, our analysis shows that a critical feature of the model is that the dynamic strength of child fragments must be highly variable and not always greater than the strength of the parent. This seems to be more important than any inverse correlation between fragment size and strength. In other words, the first fragmentation event must produce a variety of scenarios that include (a) one or both child fragments being weaker than the original fragment, resulting in immediate further break‐up; and (b) one or both child fragments being substantially stronger than the original fragment, and hence not undergoing any subsequent fragmentation. The importance of this aspect of the model suggests that alternative fragmentation models that account for heterogeneous internal strength (e.g., Wheeler et al., 2018) may also be very successful at replicating crater clusters on Mars.

The dimensionless lift coefficient (*C*_*L*_, Equation 3) that relates the change of trajectory angle during flight to the differential force of the air flowing around the meteoroid is a poorly constrained model parameter that is often either neglected (Register et al., 2017; Wheeler et al., 2017, 2018) or assumed to be a very small value (e.g., *C*_*L*_ < 10^−3^; Passey & Melosh, 1980). While this parameter has very little influence on most cluster characteristics, a good match to the observed distribution of cluster aspect ratios requires a lift coefficient ∼10^−2^. This new constraint on the lift coefficient may be useful for improving predictions of meteorite strewn fields that are critical for successful recovery efforts on Earth following large fireballs.

The optimal fragment separation coefficient (*C*_*S*_ ∼1–2, Equation 9), which relates the separation speed of fragments to the along‐trajectory speed at break‐up, is substantially larger than the commonly used estimate of *C*_*S*_ ≈ 0.13 based on theoretical consideration of bow‐shock interaction as well as shock physics modeling of interaction between two identical fragments and atmosphere (Artemieva & Shuvalov, 2001). It is closer to, but larger still than the estimate of *C*_*S*_ ≈ 0.67 obtained from shock physics modeling of atmospheric interaction of a fragmented chondritic asteroid comprising 13–27 identical fragments (Artemieva & Shuvalov, 2001). It is also at the upper bound of previous empirical estimates based on the separation of craters in terrestrial strewn fields and martian clusters (Passey & Melosh, 1980; Popova et al., 2007) and venusian crater fields (Herrick & Phillips, 1994; Korycansky & Zahnle, 2005).

On the other hand, the implied separation speeds of our successful model are broadly consistent with observations of terrestrial bolides (Popova et al., 2007). For a typical break‐up altitude on Mars of 10 km (where air density is approximately equivalent to 40 km altitude on Earth), the separation speeds of equal sized fragments implied by our best‐fit model are in the range 0.2%–0.3% of the pre‐entry velocity. This is comparable with the median separation velocity inferred from detailed observations of the Morávka meteorite fall, where break‐up occurred at an altitude of 30–45 km altitude, although maximum relative separation speeds of up to 1.5% were observed (Boroviĉka & Kalenda, 2003).

Taken together, the implied high separation speed may indicate that a supplementary separation mechanism, such as explosive release of volatiles or volumetric evaporation (Nemtchinov et al., 1999; Popova et al., 2007), acts to push the fragments apart more quickly than bow‐shock interaction alone.

### 4.2 Implications for the Rate of Small Impacts on Mars

A subset of 44 recent impact sites imaged before and after impact by Mars Reconnaissance Orbiter's Context Camera have been used to derive an estimate of the current rate of small impacts on Mars that accounts for the area and time period over which detection was possible (Daubar et al., 2013). A later update using 110 impacts and an updated area‐time factor resulted in a nearly identical rate (Daubar et al., 2014). The estimated cumulative production rate of craters with an effective diameter larger than 10 m (*N*_ > 10_) is 4.06 × 10^−7^ craters per km^2^ per year, which is a factor of three to five lower than the crater production models of (Hartmann, 2005) and (Neukum et al., 2001), respectively, based on the formation rate of much larger lunar craters.

The impactor mass‐frequency distribution employed in the Monte Carlo model used here is based on fireball observations on Earth (Bland & Artemieva, 2006; Halliday et al., 1996). Comparison between the implied cratering rate of the best‐fit model and the inferred cratering rate from observations therefore represents an independent test of the small impact rate on Mars (Williams et al., 2014). The best‐fit model can be used to estimate the small impact cratering rate on Mars if two important parameters are known: (a) the Earth/Mars impact flux ratio *R*_*ME*_; and (b) the proportion of impactors that are of asteroidal origin *f*_*A*_ (i.e., are not cometary). As discussed above, cometary meteoroids are not considered in the Monte Carlo model because they very rarely form craters or clusters in our model. This implies that the fraction of cometary impacts at Mars is not possible to constrain with observations of this sort.

**Figure 11:**
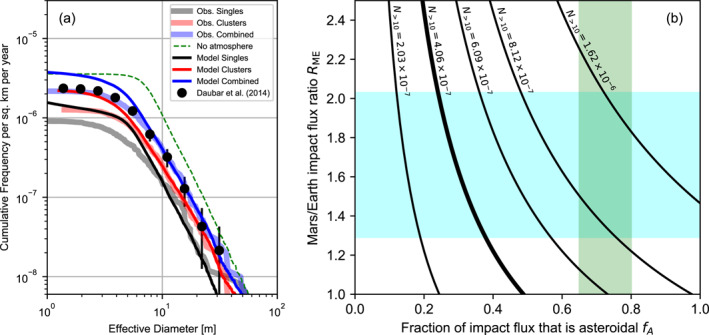
The estimated rate of small impacts on Mars. (a) A comparison of the simulated cumulative size‐frequency distribution of small impact craters and crater clusters on Mars per square km per year with an estimate based on observed craters and clusters (Daubar et al., 2014) (D2014). Also shown is the corresponding model outcome for no atmosphere. (b) The predicted cumulative number of craters or clusters with effective diameter larger than 10 m as a function of the fraction of the impact flux that is asteroidal and the Mars/Earth impact flux ratio. The symbols in (a) show the D2014 estimated annual cumulative size‐frequency distribution of small craters. The thick curves in (a) show the CFD of craters and clusters from Neidhart et al. (2021) and Daubar et al. (2022) scaled to match the D2014 cumulative cratering rate for craters larger than 10 m. The thin curves in (a) show the CFD of synthetic craters and clusters scaled to match the observed CFD for craters/clusters with an effective diameter larger than 10‐m, which requires a *f*_*A*_‐*R*_*ME*_ combination denoted by the thick line in (b). In (b) the blue shaded region denotes the range of proposed Mars/Earth impact flux ratios; the green region indicates an approximate estimate of the fraction of impact flux that is asteroidal, based on fireball observations.

A predicted production rate of craters or clusters on Mars with an effective diameter greater than 10 m, *N*_ > 10_, is obtained from the cratering rate from the best‐fit model times the product *f*_*A*_ *R*_*ME*_ (Figure 11b). To match the cratering rate inferred from observations of *N*_ > 10_ = 4.06 × 10^−7^ (Daubar et al., 2014) requires *f*_*A*_ *R*_*ME*_ ≈ 0.5, shown by the thick line in Figure 11b. When scaled by this factor, a comparison between the predicted cumulative size‐frequency distribution of craters/clusters and the observed cumulative size‐frequency distribution of craters/clusters in the recent catalog (Daubar et al., 2022) shows good agreement for craters and clusters larger than 8‐m effective diameter (Figure 11).

The slope of the cumulative size‐frequency distribution CSFD of the observed craters and clusters is 2.39 ± 0.07 (2.38 ± 0.05 for clusters alone) for effective diameters >8 m. The equivalent CSFD slope for the model is 2.52 ± 0.04 (2.41 ± 0.06) in reasonable agreement with observations, particularly with respect to clusters. An equivalent Monte Carlo simulation with no atmosphere shows the important effect of atmospheric filtering on the CSFD slope (dashed line, Figure 11). The CSFD slope of the synthetic crater population with no atmosphere is 2.94 ± 0.07, which is more than 15%–20% steeper than the CSFDs of the best‐fit Monte Carlo simulation and observed craters. The effects of atmospheric entry reduce the cumulative number of craters with effective diameter greater than 10 m by more than a factor of two relative to the airless case.

Estimates of the Mars/moon impact flux ratio based on dynamical models range from 2.04 to 3.20 (Ivanov, 2001; Le Feuvre & Wieczorek, 2011; Marchi, 2021). Adopting an Earth/Moon impact flux ratio of 1.58 (Le Feuvre & Wieczorek, 2011) implies a Mars/Earth flux ratio in the range 1.3–2 (blue region; Figure 11b).

Terrestrial fireball observations suggest that the proportion of the impactor flux in the 1–10 kg mass range that is of asteroidal origin may be in the range 65%–80% (Ceplecha et al., 1998; Halliday et al., 1996). Assuming that the composition of the impactor flux is the same at Mars and Earth, the combined constraints on *R*_*ME*_ and *f*_*A*_ suggest a Mars small crater production rate that is 1.5–4 times higher than the observation‐based estimate of Daubar et al. (2014), which could reflect the number of current impacts missing from the observational data set. This range encompasses the production rate proposed by Williams et al. (2014), based on a similar modeling approach, and the crater production model of Hartmann (2005).

On the other hand, to match the cratering rate inferred from observations of *N*_ > 10_ = 4.06 × 10^−7^ (Daubar et al., 2014) requires either a Mars‐Earth impact flux ratio less than one or a much higher proportion of cometary (low density) impactors at Mars than the proportion inferred at Earth.

### 4.3 Implications for the Seismic Detection of Crater Clusters on Mars

**Figure 12:**
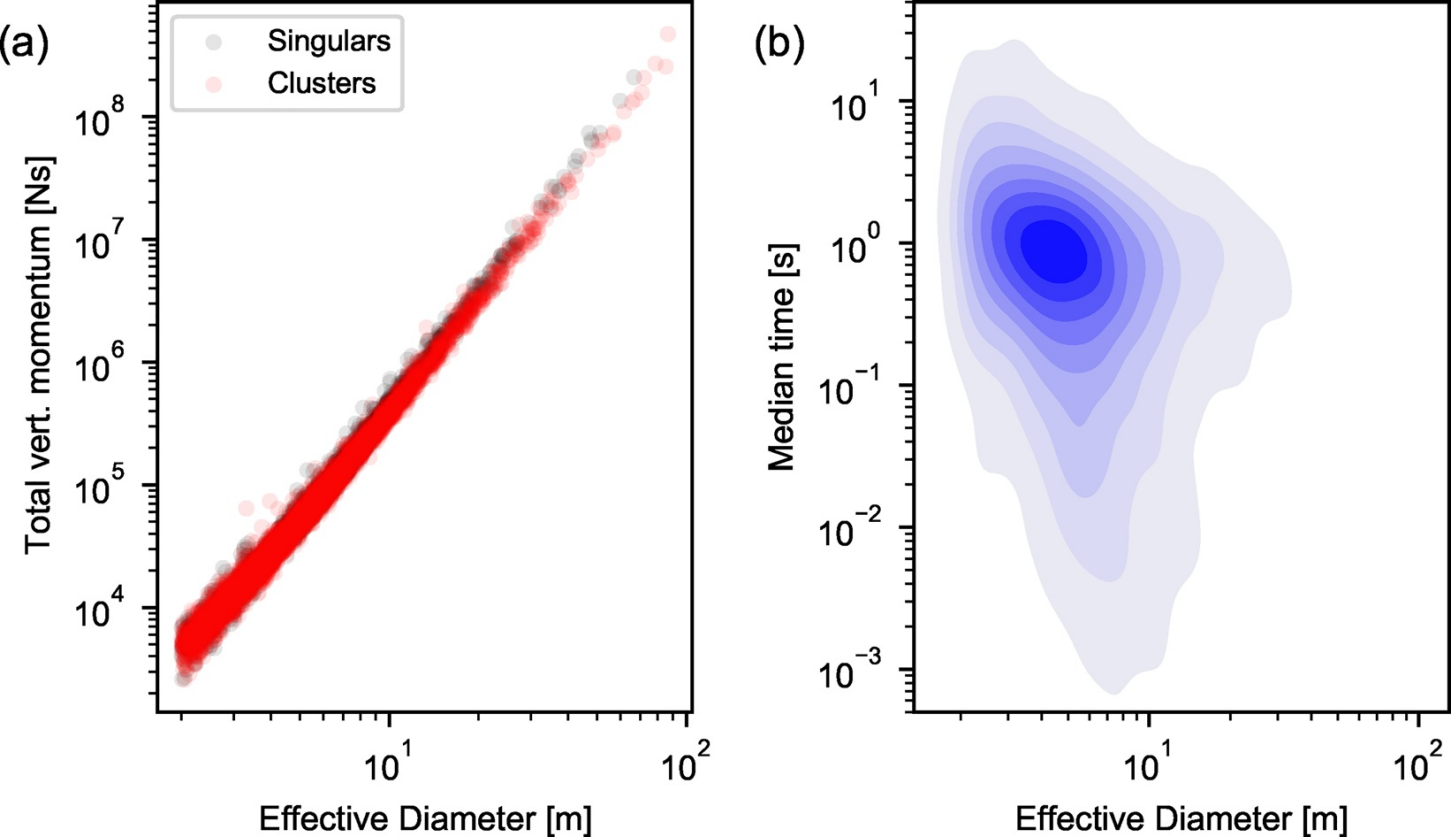
The total vertical impactor momentum for single craters and crater clusters (a) and the median time separation between fragment impacts (b) as a function of effective crater diameter for the distribution of impact scenarios from the best‐fit simulation.

A well calibrated model of crater cluster formation allows us to empirically relate impactor properties of interest to cluster properties. In future, this may be useful for interpreting newly formed impacts on Mars and their seismic detectability. For example, as the effective diameter of the crater correlates very well with the vertical component of the total momentum of all fragments impacting the ground (Figure 12a), it serves as an excellent predictor of the total seismic moment of the impact, regardless of whether it forms a cluster or a single crater (Schmerr et al., 2019; Wójcicka et al., 2020). Our results also provide insight into the median time interval between fragments striking the ground (Figure 12b), which is important for the frequency content of impact generated seismic signals (Schmerr et al., 2019). For the size range of impacts in our model, which are those most relevant for possible seismic detection by NASA's InSight lander (Daubar et al., 2018), the time interval between fragment impacts are typically less than one second, although for some highly dispersed clusters the time interval can be as long as 10 s.

**Figure 13:**
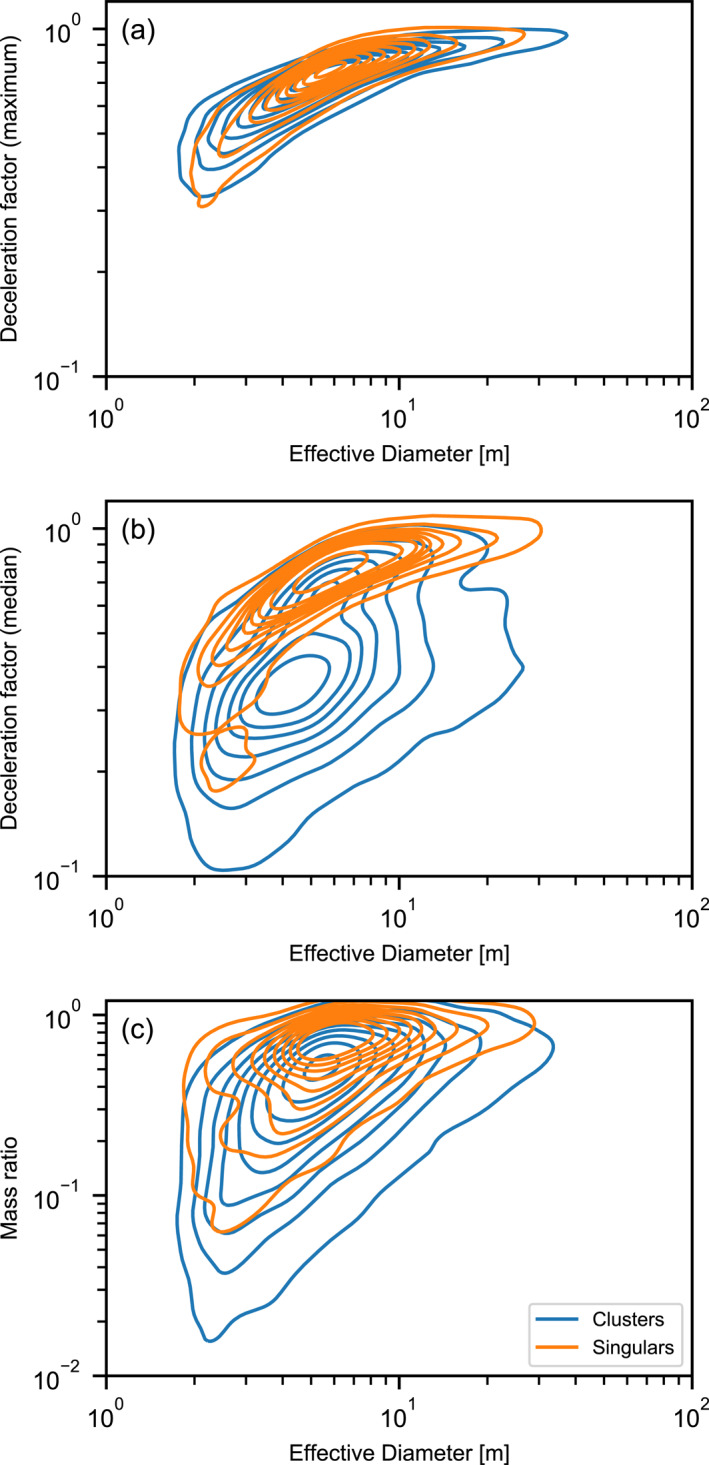
A comparison of deceleration and ablation between cluster and single‐crater impacts. The ratio of the maximum fragment velocity to the pre‐entry velocity (a); the ratio of the median fragment velocity to the pre‐entry velocity (b); and the ratio of the final mass to the pre‐entry mass (c) for both cluster‐forming and crater‐forming impacts as a function of effective diameter.

It is also evident from analysis of our model results that a cluster and a single crater of the same effective diameter can differ dramatically in terms of their initial meteoroid properties (Figure 13) because fragmented meteoroids undergo substantially greater deceleration and ablation in the atmosphere. While the fragments that produce the largest craters within a cluster undergo the same degree of deceleration as meteoroids that form single craters (Figure 13a), the more abundant smaller fragments are decelerated much more dramatically (Figure 13b). Consequently, the total meteoroid mass that strikes the ground to form a crater cluster of a given effective diameter is typically a much smaller proportion of the initial meteoroid mass than is the case for the equivalent size single crater (Figure 13c). This also implies that newly formed crater clusters represent excellent targets for meteorite detection and recovery on Mars.

## 5 Conclusions

The Separate Fragments Model of meteoroid fragmentation during atmospheric entry involves the sequential, pair‐wise division of the meteoroid into numerous fragments and their subsequent separation, deceleration and ablation before striking the ground. In this paper, we have shown that this model is able to reproduce the observed population of crater clusters on Mars in terms of all their observed characteristics. An important feature of the successful fragmentation model is that each fragmentation event is able to produce child fragments with highly variable dynamic strength that can be both much stronger and much weaker than the strength of the parent fragment.

Our work assumed a log‐uniform distribution of meteoroid strengths between an upper and lower bound. To best reproduce the observed distribution of crater clusters, including the ratio of clusters to single craters, required a meteoroid strength distribution with a minimum strength of 10–90 kPa, a maximum strength of 3–6 MPa and a median strength of 0.2–0.5 MPa. This range is very consistent with the inferred dynamic strengths of terrestrial fireball meteoroids.

Optimization of the model also places new constraints on the separation speed of fragments after break‐up and the lift coefficient that relates the change of trajectory angle during flight to the differential force of the air flowing around the meteoroid fragments. To match the observed distribution of cluster aspect ratios requires a lift coefficient ∼10^−2^, which is several times higher than previous estimates. Similarly, to match the observed median separation distance between crater pairs (dispersion) requires fragment separation speeds of 0.2%–0.3% of the pre‐entry meteoroid velocity. Such speeds are at the upper end of previous model estimates but are comparable with the median separation velocity inferred from detailed observations of terrestrial meteorite falls.

The calibrated model suggests that the rate of small impacts on Mars is 1.5–4 times higher that current observation‐based estimates (Daubar et al., 2013, 2014). Lower small crater production rates require a significantly lower Mars/Earth flux ratio than current estimates based on dynamical models (Ivanov, 2001; Le Feuvre & Wieczorek, 2011) or an unexpectedly high (>50%) proportion of low‐density, cometary meteoroids among the Mars impactor flux. It therefore seems likely that a substantial proportion of meter‐to‐decameter craters forming on Mars are not being detected with current orbital imaging techniques.

The success of the Separate Fragments model in reproducing the diversity of observed clusters suggests that future efforts to infer impactor properties from observed cluster characteristics by inversion may be fruitful. The formation of crater clusters is favored by low‐to‐moderate impact speeds, low meteoroid strengths and relatively steep trajectories to the surface. Newly formed crater clusters represent excellent targets for meteorite detection and recovery on Mars.

## Data Availability

The implementation of the Separate Fragments Model used here is published as Schwarz et al. (2022). The Monte Carlo code, post‐processing code and synthetic data are published as Collins et al. (2022). The observational data is published as: Neidhart et al. (2021).

## References

[jgre21960-bib-0001] Artemieva, N. A. , & Shuvalov, V. V. (2001). Motion of a fragmented meteoroid through the planetary atmosphere. Journal of Geophysical Research, 106(E2), 3297–3309. 10.1029/2000JE001264

[jgre21960-bib-0002] Artemieva, N. A. , & Shuvalov, V. V. (2016). From Tunguska to Chelyabinsk via Jupiter. Annual Review of Earth and Planetary Sciences, 44(1), 37–56. 10.1146/annurev-earth-060115-012218

[jgre21960-bib-0003] Baldwin, B. , & Sheaffer, Y. (1971). Ablation and breakup of large meteoroids during atmospheric entry. Journal of Geophysical Research, 76(19), 4653–4668. 10.1029/JA076i019p04653

[jgre21960-bib-0004] Bland, P. A. , & Artemieva, N. A. (2006). The rate of small impacts on Earth. Meteoritics & Planetary Sciences, 41(4), 607–631. 10.1111/j.1945-5100.2006.tb00485.x

[jgre21960-bib-0005] Boroviĉka, J. , & Kalenda, P. (2003). The Morávka meteorite fall: 4. Meteoroid dynamics and fragmentation in the atmosphere. Meteoritics & Planetary Sciences, 38(7), 1023–1043. 10.1111/j.1945-5100.2003.tb00296.x

[jgre21960-bib-0006] Borovička, J. , Popova, O. , & Spurný, P. (2019). The Maribo CM2 meteorite fall—Survival of weak material at high entry speed. Meteoritics & Planetary Sciences, 54(5), 1024–1041. 10.1111/maps.13259

[jgre21960-bib-0007] Borovička, J. , Spurný, P. , & Shrbený, L. (2020). Two strengths of ordinary chondritic meteoroids as derived from their atmospheric fragmentation modeling. The Astronomical Journal, 160(1), 42. 10.3847/1538-3881/ab9608

[jgre21960-bib-0008] Britt, D. T. , & Consolmagno, G. J. S. J. (2003). Stony meteorite porosities and densities: A review of the data through 2001. Meteoritics & Planetary Sciences, 38(8), 1161–1180. 10.1111/j.1945-5100.2003.tb00305.x

[jgre21960-bib-0009] Brykina, I. G. , & Bragin, M. D. (2020). On models of meteoroid disruption into the cloud of fragments. Planetary and Space Science, 187, 104942. 10.1016/j.pss.2020.104942

[jgre21960-bib-0010] Ceplecha, Z. , Borovička, J. , Elford, W. G. , ReVelle, D. O. , Hawkes, R. L. , Porubčan, V. , & Šimek, M. (1998). Meteor phenomena and bodies. Space Science Reviews, 84(3), 327–471. 10.1023/A:1005069928850

[jgre21960-bib-0011] Chappelow, J. E. , & Sharpton, V. L. (2005). Influences of atmospheric variations on Mars’s record of small craters. Icarus, 178(1), 40–55. 10.1016/j.icarus.2005.03.010

[jgre21960-bib-0012] Chyba, C. F. , Thomas, P. J. , & Zahnle, K. J. (1993). The 1908 Tunguska explosion: Atmospheric disruption of a stony asteroid. Nature, 361(6407), 40–44. 10.1038/361040a0

[jgre21960-bib-0013] Collins, G. S. , Schwarz, D. , Coleman, M. , & Newland, E. L. (2022). Meteoroid fragmentation in the martian atmosphere and the formation of crater clusters: Source code and data. Zenodo. 10.5281/zenodo.6566345 PMC9541127 36247718

[jgre21960-bib-0014] Daubar, I. J. , Banks, M. E. , Schmerr, N. C. , & Golombek, M. P. (2019). Recently formed crater clusters on Mars. Journal of Geophysical Research: Planets, 124(4), 958–969. 10.1029/2018JE005857 PMC6999777 32021742

[jgre21960-bib-0015] Daubar, I. J. , Dundas, C. M. , McEwen, A. S. , Gao, A. , Wexler, D. , Piqueux, S. , et al. (2022). New craters on Mars: An updated catalog. Journal of Geophysical Research: Planets, 127, e2021JE007145. 10.1029/2021JE007145

[jgre21960-bib-0016] Daubar, I. J. , Lognonné, P. , Teanby, N. A. , Miljkovic, K. , Stevanović, J. , Vaubaillon, J. , et al. (2018). Impact‐seismic investigations of the InSight mission. Space Science Reviews, 214(8), 132. 10.1007/s11214-018-0562-x

[jgre21960-bib-0017] Daubar, I. J. , McEwen, A. S. , Byrne, S. , Kennedy, M. R. , & Ivanov, B. (2013). The current martian cratering rate. Icarus, 225(1), 506–516. 10.1016/j.icarus.2013.04.009

[jgre21960-bib-0018] Daubar, I. J. , McEwen, A. S. , Byrne, S. , Kreslavsky, M. , Saper, L. , & Kennedy, M. R. (2014). New dated impacts on Mars and an updated current cratering rate. In Eighth International Conference on Mars (Vol. 1791, pp. 1007). Lunar and Planetary Institute. Retrieved from https://ui.adsabs.harvard.edu/abs/2014LPICo1791.1007D

[jgre21960-bib-0019] Fernando, B. , Wójcicka, N. , Maguire, R. , Stähler, S. C. , Stott, A. E. , Ceylan, S. , et al. (2021). Seismic constraints from a Mars impact experiment using InSight and Perseverance. Nature Astronomy, 1(1), 59–64. 10.1038/s41550-021-01502-0

[jgre21960-bib-0020] Forget, F. , Hourdin, F. , Fournier, R. , Hourdin, C. , Talagrand, O. , Collins, M. , et al. (1999). Improved general circulation models of the Martian atmosphere from the surface to above 80 km. Journal of Geophysical Research, 104(E10), 24155–24175. 10.1029/1999JE001025

[jgre21960-bib-0021] Halliday, I. , Griffin, A. A. , & Blackwell, A. T. (1996). Detailed data for 259 fireballs from the Canadian camera network and inferences concerning the influx of large meteoroids. Meteoritics & Planetary Sciences, 31(2), 185–217. 10.1111/j.1945-5100.1996.tb02014.x

[jgre21960-bib-0022] Hartmann, W. K. (2005). Martian cratering 8: Isochron refinement and the chronology of Mars. Icarus, 174(2), 294–320. 10.1016/j.icarus.2004.11.023

[jgre21960-bib-0023] Hartmann, W. K. , Daubar, I. J. , Popova, O. , & Joseph, E. C. S. (2018). Martian cratering 12. Utilizing primary crater clusters to study crater populations and meteoroid properties. Meteoritics & Planetary Sciences, 53(4), 672–686. 10.1111/maps.13042

[jgre21960-bib-0024] Herrick, R. R. , & Phillips, R. J. (1994). Effects of the Venusian atmosphere on incoming meteoroids and the impact crater population. Icarus, 112(1), 253–281. 10.1006/icar.1994.1180

[jgre21960-bib-0025] Hills, J. G. , & Goda, M. P. (1993). The fragmentation of small asteroids in the atmosphere. The Astronomical Journal, 105, 1114–1144. 10.1086/116499

[jgre21960-bib-0026] Holsapple, K. A. (1993). The scaling of impact processes in planetary sciences. Annual Review of Earth and Planetary Sciences, 21(1), 333–373. 10.1146/annurev.ea.21.050193.002001

[jgre21960-bib-0027] Ivanov, B. A. (2001). Mars/Moon cratering rate ratio estimates. Space Science Reviews, 96(1), 87–104. 10.1007/978-94-017-1035-0_4

[jgre21960-bib-0028] Ivanov, B. A. , Melosh, H. J. , McEwen, A. S. , & HiRISE Team . (2009). Small impact crater clusters in high resolution HiRISE images—II. 40th lunar and planetary science conference (Vol. 1410). Retrieved from https://ui.adsabs.harvard.edu/abs/2009LPI.40.1410I

[jgre21960-bib-0029] Kite, E. S. , Williams, J.‐P. , Lucas, A. , & Aharonson, O. (2014). Low palaeopressure of the martian atmosphere estimated from the size distribution of ancient craters. Nature Geoscience, 7(5), 335–339. 10.1038/ngeo2137

[jgre21960-bib-0030] Korycansky, D. G. , & Zahnle, K. J. (2005). Modeling crater populations on Venus and Titan. Planetary and Space Science, 53(7), 695–710. 10.1016/j.pss.2005.03.002

[jgre21960-bib-0031] Le Feuvre, M. , & Wieczorek, M. A. (2011). Nonuniform cratering of the Moon and a revised crater chronology of the inner Solar System. Icarus, 214(1), 1–20. 10.1016/j.icarus.2011.03.010

[jgre21960-bib-0032] Malin, M. C. , Edgett, K. S. , Posiolova, L. V. , McColley, S. M. , & Dobrea, E. Z. N. (2006). Present‐day impact cratering rate and Contemporary gully activity on Mars. Science, 314(5805), 1573–1577. 10.1126/science.1135156 17158321

[jgre21960-bib-0033] Marchi, S. (2021). A new martian crater chronology: Implications for Jezero crater. The Astronomical Journal, 161(4), 187. 10.3847/1538-3881/abe417

[jgre21960-bib-0034] McMullan, S. , & Collins, G. S. (2019). Uncertainty quantification in continuous fragmentation airburst models. Icarus, 327, 19–35. 10.1016/j.icarus.2019.02.013

[jgre21960-bib-0035] Millour, E. , Forget, F. , Spiga, A. , Vals, M. , Zakharov, V. , Montabone, L. , & The MCD development team (2018). The Mars climate database (Version 5.3). In Scientific workshop: From Mars express to ExoMars. ESAC. Retrieved from https://www.cosmos.esa.int/documents/1499429/1583871/Millour_E.pdf

[jgre21960-bib-0036] Neidhart, T. , Sansom, E. , Miljković, K. , Daubar, I. , Eschenfelder, J. , & Collins, G. (2021). New crater clusters on Mars. Zenodo. 10.5281/zenodo.5760176

[jgre21960-bib-0037] Nemtchinov, I. V. , Kuzmicheva, M. Y. , Shuvalov, V. V. , Golub’, A. P. , Popova, O. P. , Kosarev, I. B. , & Borovička, J. (1999). Šumava meteoroid ‐ Was it a small comet? In J. Svoren , E. Pitch , & H. Rickman (Eds.), Evolution and source regions of asteroids and Comets (Vol. 173, pp. 51–56). Tatranska Lomnica: Astron. Inst. Slovak Acad. Sci. 10.1017/s0252921100031237

[jgre21960-bib-0038] Neukum, G. , Ivanov, B. A. , & Hartmann, W. K. (2001). Cratering records in the inner solar system in relation to the lunar reference system. Space Science Reviews, 96(1–4), 55–86. 10.1023/A:1011989004263

[jgre21960-bib-0039] Passey, Q. R. , & Melosh, H. J. (1980). Effects of atmospheric breakup on crater field formation. Icarus, 42(2), 211–233. 10.1016/0019-1035(80)90072-X

[jgre21960-bib-0040] Popova, O. , Borovička, J. , Hartmann, W. K. , Spurný, P. , Gnos, E. , Nemtchinov, I. , & Trigo‐Rodríguez, J. M. (2011). Very low strengths of interplanetary meteoroids and small asteroids. Meteoritics & Planetary Sciences, 46(10), 1525–1550. 10.1111/j.1945-5100.2011.01247.x

[jgre21960-bib-0041] Popova, O. , Hartmann, W. K. , Nemtchinov, I. V. , Richardson, D. C. , & Berman, D. C. (2007). Crater clusters on Mars: Shedding light on martian ejecta launch conditions. Icarus, 190(1), 50–73. 10.1016/j.icarus.2007.02.022

[jgre21960-bib-0042] Popova, O. , Nemtchinov, I. , & Hartmann, W. K. (2003). Bolides in the present and past martian atmosphere and effects on cratering processes. Meteoritics & Planetary Sciences, 38(6), 905–925. 10.1111/j.1945-5100.2003.tb00287.x

[jgre21960-bib-0043] Register, P. J. , Mathias, D. L. , & Wheeler, L. F. (2017). Asteroid fragmentation approaches for modeling atmospheric energy deposition. Icarus, 284, 157–166. 10.1016/j.icarus.2016.11.020

[jgre21960-bib-0044] Schmerr, N. C. , Banks, M. E. , & Daubar, I. J. (2019). The seismic signatures of recently formed impact craters on Mars. Journal of Geophysical Research: Planets, 124(11), 3063–3081. 10.1029/2019JE006044 32021742 PMC6999777

[jgre21960-bib-0045] Schwarz, D. , Collins, G. S. , Newland, E. L. , & Coleman, M. (2022). Fragment‐cloud model. Zenodo. 10.5281/zenodo.6806015

[jgre21960-bib-0046] Shoemaker, E. M. (1961). Interpretation of lunar craters. In Z. Kopal (Ed.), Physics and astronomy of the moon (pp. 283–359). Academic Press. 10.1016/B978-1-4832-3240-9.50012-2

[jgre21960-bib-0047] Weibull, W. (1951). A statistical distribution function of wide applicability. Journal of Applied Mechanics, 18(3), 293–297. 10.1115/1.4010337

[jgre21960-bib-0048] Wheeler, L. F. , Mathias, D. L. , Stokan, E. , & Brown, P. G. (2018). Atmospheric energy deposition modeling and inference for varied meteoroid structures. Icarus, 315, 79–91. 10.1016/j.icarus.2018.06.014

[jgre21960-bib-0049] Wheeler, L. F. , Register, P. J. , & Mathias, D. L. (2017). A fragment‐cloud model for asteroid breakup and atmospheric energy deposition. Icarus, 295, 149–169. 10.1016/j.icarus.2017.02.011

[jgre21960-bib-0050] Williams, J.‐P. , Pathare, A. V. , & Aharonson, O. (2014). The production of small primary craters on Mars and the Moon. Icarus, 235, 23–36. 10.1016/j.icarus.2014.03.011

[jgre21960-bib-0051] Wójcicka, N. , Collins, G. S. , Bastow, I. D. , Teanby, N. A. , Miljković, K. , Rajšić, A. , et al. (2020). The seismic moment and seismic efficiency of small impacts on Mars. Journal of Geophysical Research: Planets, 125(10). 10.1029/2020JE006540

